# Morphometry of the hippocampal microvasculature in post-stroke and age-related dementias

**DOI:** 10.1111/nan.12085

**Published:** 2014-03-13

**Authors:** M J C Burke, L Nelson, J Y Slade, A E Oakley, A A Khundakar, R N Kalaria

**Affiliations:** Centre for Brain Ageing and Vitality, Institute for Ageing and Health, Newcastle University, Campus for Ageing & VitalityNewcastle upon Tyne, UK

**Keywords:** Alzheimer's disease, hippocampus, microvessel, post-stroke dementia, stroke, vascular dementia

## Abstract

**Background:**

Optimal vascular function is vital for prevention of dementia. We hypothesized that elderly post-stroke survivors who preserve cognitive function show unperturbed cerebral microvasculature compared with those who develop dementia.

**Methods:**

Using stereological spherical probe software, we compared the length density (Lv, cumulative vessel length per unit tissue volume) of hippocampal microvessels in *post mortem* brain tissue from post-stroke survivors, Alzheimer's disease (AD), vascular dementia (VaD) and normal ageing control subjects. We also assessed microvessel diameters in the same subjects. Microvessels were identified by markers of endothelial cells (glucose transporter 1; GLUT1), basement membrane (collagen IV; COL4) and smooth muscle cell α-actin (SMA).

**Results:**

We found increased Lv of both GLUT1 and COL4 immunostained microvessels (*P* < 0.05) in the hippocampal CA1 region of post-stroke demented (PSD) and AD cases compared with post-stroke nondemented (PSND), control and VaD subjects. However, no changes were apparent in the CA2 region. We also noted significant increase in Lv in the entorhinal cortex of AD compared with PSND and PSD subjects. The mean diameter of microvessels was decreased in PSD, compared with PSND, as well as in AD and VaD compared with controls. Cumulative frequency analysis showed PSND subjects to have significantly greater proportion of microvessels with diameters, ranging from 7 to 12 μm.

**Conclusions:**

An increase in microvascular Lv in AD and PSD suggests either an increase in angiogenesis or the formation of newer microvessel loops in response to cerebral hypoperfusion. The decreased vessel diameters found in AD and VaD suggests increased vasoconstriction in dementia.

## Introduction

Previous epidemiological studies have shown that vascular disease plays a key role in the progression of dementia 1,2, inclusive of cardiovascular risk factors, such as increased blood pressure 3, atrial fibrillation 4, diabetes mellitus 5 and other vascular disease-related factors 6. Magnetic resonance imaging (MRI) studies using arterial spin labelling, have shown a reduction in cerebral blood flow (CBF) in the temporal lobe associated with dementia in Alzheimer's disease (AD) 7 and in post-stroke survivors 8. In these scenarios, the decrease in CBF is thought to result from neurodegeneration, whereby the cerebral microvasculature is modulated due to lower demand for glucose and oxygen. However, an alternative view has also been proposed, in which case the decrease in CBF may be the cause or instigator of neurodegeneration 9. Consistent with this latter hypothesis, we recently proposed there is a vascular basis for neuronal atrophy and likely neurodegeneration in post-stroke demented (PSD) subjects without significant AD pathology 10.

The role of the cerebral microvasculature in age-related dementias continues to be unclear. Using different methods of assessment, previous studies in dementia have suggested microvessel density, is decreased in dementia, especially in AD 11–13, whereas others claim it is increased 14–16. Some investigators have also demonstrated narrowing of microvessels in the CA1 of the hippocampus and entorhinal cortex (EC) of AD subjects 14,17. Differences in the methods of analysis and brain regions investigated may explain the variable results. However, studies in AD have indicated that there is an up-regulation or modulation of pro-angiogenic proteins and vascular growth factors 18, which may increase the density of the microvasculature 19–21. Studies involving transgenic (Tg) mice models, which overexpress the amyloid precursor protein (APP) to simulate AD pathology, have reported decreased angiogenesis in the presence of amyloid β peptides 22. While a study using the Tg2576 mouse model found increased microvessel density and extensive disruption to tight junctions, leading to a new hypothesis of amyloidogenesis 23. Additionally, a corrosion cast model of vasculature in an AD mouse model has suggested an increase in density, as well as degeneration, of vessels 24. Conversely, other studies, in an aged APP/PS1 mouse model, have shown no change in microvessel density 25.

To clarify these issues, we examined *post mortem* tissue taken from the hippocampal formation to assess whether microvascular morphology, specifically length density (L_v_) and diameter, was affected in different dementias, placing particular emphasis on PSD. The CA1 subfield was assessed due to its importance in relation to the onset of dementia and specific susceptibility to both increased AD-like pathology, risk of ischaemia and hypoperfusion 26. L_v_ measurements were conducted using a spherical probe 27 and the diameter of cerebral microvessels were assessed using software that had been developed to measure vessel diameter and perivascular space 28. It is hypothesized that PSD cases will exhibit reduced microvascular density and diameter, compared with post-stroke nondemented (PSND). Preliminary analysis from this study was presented in abstract form at the 112th British Neuropathological Society Meeting, January 2011 29.

## Materials and methods

### Subjects and clinical features

*Post mortem* brain tissue was obtained from PSD and PSND subjects 30. AD and vascular dementia (VaD) subjects and similar age controls were included for comparison. The demographic details of the different subjects are presented in Table 1. The post-stroke subjects were enrolled in the prospective Cognitive Function After Stroke (CogFAST) study 30,31. Stroke patients aged ≥75 years were selected on the basis that they were not demented 3 months after stroke and did not exhibit disabilities that would prevent them from completing cognitive testing. They received annual clinical assessments and a neuropsychological test battery from baseline, including the Cognitive Drug Research (CDR) battery, the Mini-Mental State Exam (MMSE) and the Cambridge Assessment of Mental Disorders in the Elderly (CAMCOG), which generated subscores for various cognitive domains, including memory and executive function 30,32.

**Table 1:** Demographic details and dementia type in the subjects

	Controls	PSND	PSD	VaD	AD
Total number of controls or cases analysed	13	23	13	15	14
Age, years Mean (range)	80.4 (72–94)	84.3 (78–94)	86.3 (80–96)	85.1 (71–97)	83.5 (70–91)
PMD, h Mean (± 2SEM)	28.0 (6.1)	45 (11.2)	47.2 (14.6)	35.0 (13.4)	59 (15.8)
MMSE score mean (range)	N/A	27.1 (24–30)	17.3 (12–24)	N/A	N/A
CAMCOG score (range)	N/A	89.1 (82–99)	62.6 (24–80)	N/A	N/A
Braak stage Mean (range)	2.4 (1–4)	2.5 (1–5)	2.6 (0–4)	2.0 (1–4)	5.3 (4–6)
CERAD	0.5 (0–1)	1.4 (0–2)	1.0 (0–3)	1.0 (0–2)	3.0 (3–3)
Mean (range)	2.2 (0–4)	2.8 (1–4)	1.6 (0–4)	1.9 (0–3)	3.7 (3–4)
Vascular pathology* (range)	NPD	12.6 (7–16)	11.9 (8–17)	13 (12–14)	N/A
Mean time from diagnosis of dementia to death (years)	–	–	2.38	4.58	4.0

* Vascular pathology scores were derived as described previously 10. For stereological analysis, six samples representing each disease type or controls were selected from the total pool and matched for age, *post mortem* interval (PMD) and fixation length.

The causes of death included bronchopneumonia, cardiac arrest and carcinoma with no particular distribution in any group. The time period (weeks) of tissue fixation was in range 8–15 weeks for all the cases.

Abbreviations: AD, Alzheimer's disease; CAMCOG, Cambridge Assessment of Mental Disorders in the Elderly; CERAD Consortium to Establish a Registry for Alzheimer's disease; MMSE, Mini-Mental State Exam; NPD, no neuropathological diagnosis; N/A, not applicable; PMD, *post mortem* delay; PSND, post-stroke nondemented; PSD, post-stroke dementia; VaD, vascular dementia.

Subjects were classified as demented if they met DSM-IIIR criteria for dementia. Controls aged >75 years were only selected if they had not been diagnosed clinically with cognitive impairment. There was no significant difference between the groups in average survival time (59.4 months) post-ischaemic injury event. Ethical approval was granted by local research ethics committees for this study (Newcastle upon Tyne Hospitals Trust, UK) and permission for *post mortem* research using brain tissue was granted for this project. All tissue was obtained from the Newcastle Brain Tissue Resource.

### Neuropathological examination

Final classification of demented subjects was assigned based on established neuropathological diagnostic criteria 33. Briefly, haematoxylin-eosin staining was used for assessment of structural integrity and infarcts, Nissl and luxol fast blue staining for cellular pattern and myelin loss, Bielschowsky's silver impregnation for CERAD rating of neuritic plaques, and tau immunohistochemistry for Braak staging of neurofibrillary tangles. A diagnosis of VaD was made when there were multiple or cystic infarcts, lacunae, microinfarcts and small vessel disease, and Braak stage <III 33. A diagnosis of AD was confirmed on evidence of significant Alzheimer's type pathology, namely a Braak stage V–VI score, a moderate–severe CERAD score and an absence of significant vascular pathology. Thal staging 34 was also performed: the hippocampal formation and medial temporal lobe were stained for amyloid (4G8 antibody) and each case was graded, dependent on staging criteria. Vascular pathology scores were derived from the presence of vascular lesions in brain areas, including the frontal lobe at the level of the olfactory bulbs, temporal lobe at level of the anterior hippocampus, and basal ganglia at level of mamillary body. Lesions including arteriolosclerosis, cerebral amyloid angiopathy, perivascular haemosiderin leakage, perivascular space dilatation in the deep and juxtacortical white matter (WM), myelin loss, and cortical micro (<0.5 cm) and large (>0.5 cm) infarcts were recorded with increasing severity resulting in greater scores 10. Control subject tissue was determined not to have had sufficient pathology to reach threshold to ascertain a diagnosis for dementia (all pathological scores are shown in Table 1)

### Immunohistochemistry

Paraffin wax-embedded human hippocampal blocks were selected based upon their proximity to a specific plane, adjacent to the anterior pole of the lateral geniculate body in the coronal plane, within the posterior section of the hippocampus. Due to limited access to tissue, as a result of working in the confines of a brain bank environment, *only one block per case* was available for analysis. For stereological analysis, 15 30-μm-thick serial sections were cut from the front of the block and mounted onto Superfrost+ slides (Fisher Scientific, Loughborough, UK). Every fifth section was then chosen so that three sections per case were selected for uniform random sampling prior to staining with each of the markers for immunohistochemistry: glucose transporter 1 (GLUT1), collagen IV (COL4) and smooth muscle α-actin (SMA). Ten-μm-thick sections were used for vessel diameter analysis and mounted on 2% 3-aminopropyltriethoxysilane (APES) slides before being stained using a standard immunohistochemical technique for COL4.

In the initial preliminary study, serially cut 10-μm-thick hippocampal sections taken from all dementia groups and controls (Table 1) were used to perform standard two-dimensional analysis of the microvasculature and labelled with the same endothelial and basement membrane markers.

**Figure 1:**
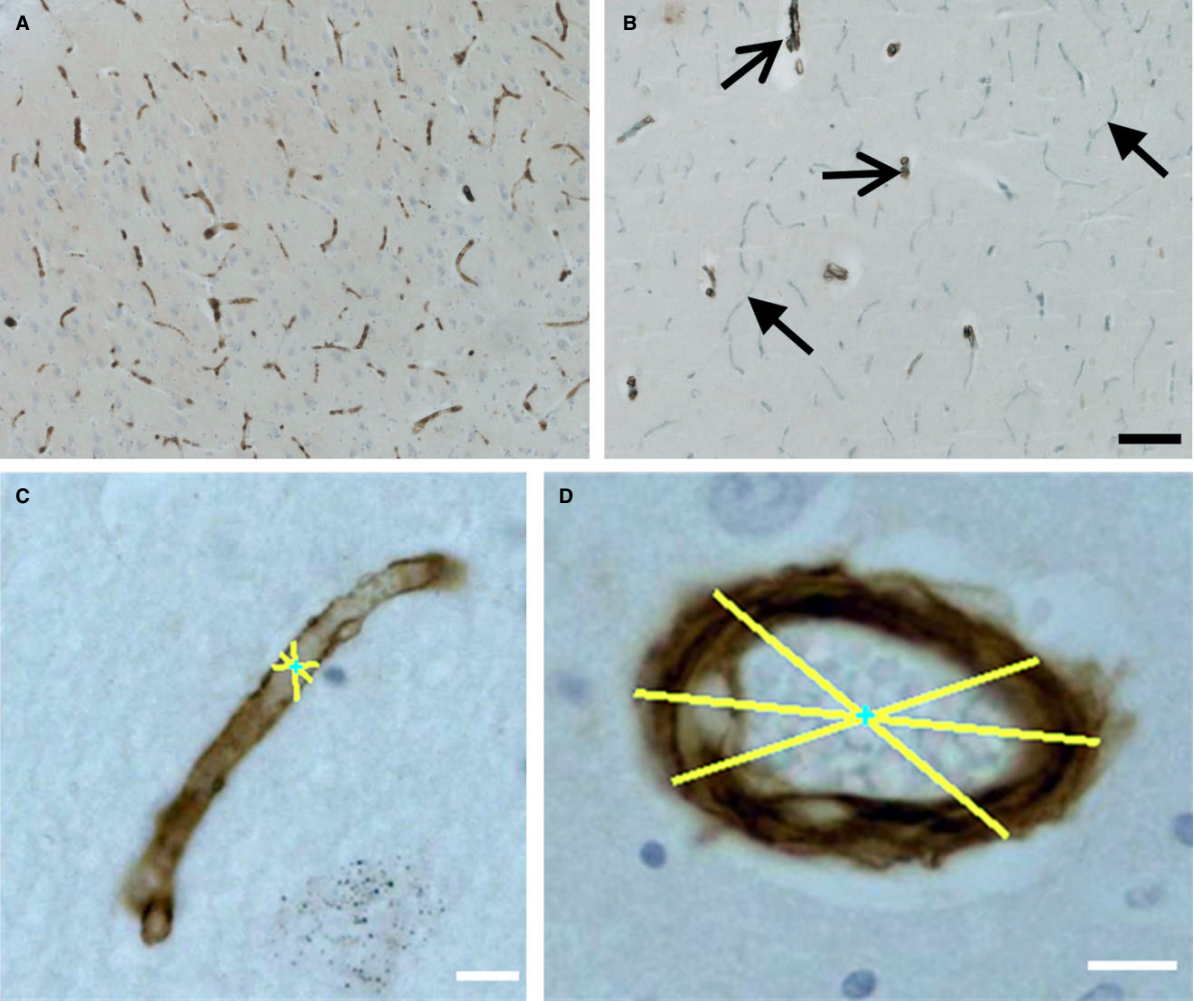
Microvasculature in the CA1 region of the hippocampal formation. (**A**) Microvessels immunostained with GLUT1 antibodies for endothelial cells. (**B**) Microvessels and arterioles stained for basement membranes for COL4 (grey, arrowhead) and smooth muscle α-actin (brown, open arrows). Note, GLUT1 stained microvessels appear discontinuous compared with COL4 (cf. **A** *vs.* **B**). (**C** and **D**) Morphometric technique used to measure vasculature diameter stained with COL4 in 10 μm sections. (**C**) A longitudinal cut vessel and (**D**) a transectionally cut vessel. Magnification bars: **B** = 100 μm; **C** and **D** = 10 μm.

Antigen heat retrieval was conducted by placing the 30-μm-thick sections in boiling 0.1 M citrate buffer for 10–15 min. The primary antibodies used were as follows: GLUT1 (ThermoScientific, Loughborough, UK, 1:200), COL4 (Sigma Aldrich, London, UK, 1:500) and SMA (Sigma Aldrich, UK, 1:1000). A protease antigen retrieval step was performed in the 10-μm-thick sections immunostained for COL4 by treating with 0.6% subtilisin A, type VIII bacterial protease (Sigma, London, UK) solution for 10 min at room temperature. Appropriate secondary antibodies were used, followed by incubation with Vectastain Avidin/Biotinylated Complex (ABC) to increase sensitivity of staining (Vector Labs, Peterborough, UK). GLUT1, SMA and COL4 (10 μm) were visualized using 3,3′-diaminobenzidine and COL4 (30 μm) with Vector SG and counterstained with haematoxylin (Figure 1).

### Stereological and image analysis: length density and vessel diameter

Initially, standard two-dimensional analysis was undertaken to assess trends in microvascular changes within various dementia groups 35. However, to strengthen the preliminary findings and discern the three-dimensional aspect of the microvasculature, an adapted stereological protocol was used.

The operators (M.J.C.B., hippocampus and L.N., EC) performed analysis blindly in order to restrict operator bias. Using the Stereologer2000 software (Stereologer, Alexandria, VA, USA), a spherical probe ‘space ball’ option was selected to measure L_v_ of microvasculature 27. The operating system was connected to a Zeiss Axiolab microscope with a motorized stage (Prior Scientific, Cambridge, UK).

In this study, a spherical probe with a diameter of 18 μm was selected to allow for section shrinkage and an appropriate guard volume. An outline was drawn denoting the area of interest, which corresponded to the relevant hippocampal subfield or cortical region at low magnification (×5). Neuronal subfields were visualized with the aid of haematoxylin counter staining. A digitally generated, equally spaced grid was overlaid and used to ensure random sampling within *x-* and *y*-axis of the reference area. A pilot study was performed to determine the number of frames required to reduce the sampling coefficient of error (CE) to a satisfactory level. Such calculations were based on the density of vessels in a particular region, with the probe size and distance between probes altered accordingly. L_v_ was calculated by counting the number of intersections between the probe and the parameter – in this instance microvasculature (ΣQ), and the area of sampling probe (ΣA) [L_v_ = 2(ΣQ/ΣA)] at ×100 magnification 27. The number of intersections was used to estimate the L_v_ for each case. As stated earlier, a lack of tissue availability prevented the full sampling of the region of interest, thus precluding the measurement of volume of the hippocampal structures and the subsequent calculation of total length using L_v_ estimates.

Images for vessel diameter analysis were taken from 10-μm-thick sections stained with COL4 at × 40 magnification using a Zeiss AX10 research-grade microscope from the CA1 region. Approximately 30 images were taken at random across the area of interest in each case. Analysis of the vessel diameter was determined using software developed to measure vessel diameter. The software was calibrated by measuring a known length from a graticule at the selected magnification of analysis 28. Length was calculated by using digital generated lines drawn by the operator across the subject matter of interest. Each vessel was measured three times and the average from these measurements was recorded as the mean vessel diameter (Figure 1**B**).

### Statistical analysis

Statistical analysis was carried out using IBM SPSS software (version 19.0). Significance was determined at *P* ≤ 0.05. The Shapiro–Wilk test was used to test for normality of stereology data, parametric tests were used to analyse hippocampal data, and nonparametric tests were used for EC data. Group means were compared using an anova and Tukey *post-hoc* test, or Kruskal–Wallis and Mann–Whitney *U*-test. Correlations were assessed using Pearson's correlation. The Komolgrov–Smirnof test was used to assess the normality of vessel diameter data, as collective data were analysed from each group. Nonparametric Kruskal–Wallis test was used to analyse data and the Mann–Whitney *U*-test was used to establish significance between groups. Cumulative frequency analysis was performed of increasing vessel diameter to determine if subpopulations of vessels were responsible for changes in mean. Comparisons between group cumulative frequencies and power analyses were carried out using Multitab 16 Statistical Software (Minitab Inc., State College, PA, USA).

## Results

### Neuropathological findings

There were no significant differences in any of the pathological staging results between the PSND and PSD cases. Significant increases (*P* ≤ 0.05) were apparent between the AD group and other disease groups for Braak stages, CERAD rating and Thal stages. The maximum Thal score that could be assigned to an individual case was stage four, due to the hippocampal formation and EC being the only regions assessed (Table 1).

### Length density assessment

Initial assessment of hippocampal microvasculature was conducted using standard two-dimensional image analysis with ImagePro 4.0 software (Mediacypernetics, Silverspring, MD, USA). Ten-micrometre-thick sections were stained with GLUT1 and the data were represented as a percentage per area of staining in a total of 78 samples in all groups (Table 1). Significant increases in percentage per area were found in GLUT1 density in AD cases in the CA1, compared with PSND and PSD (*P* = 0.011 and 0.037 respectively). A trend towards a significant decrease in GLUT1 expression was found in PSND cases, compared with controls (*P* = 0.073). A significant increase was also found in COL4 percentage per area in AD cases, compared with controls and VaD (*P* = 0.024 and *P* = 0.05 respectively). The only significant difference in CA2 was found with GLUT1, with an increase in AD compared with VaD (*P* = 0.04). Based on these preliminary data, we wished to address issues surrounding microvascular orientation, stemming from the use of two-dimensional analysis, which may have led to inaccurate conclusions. A modified stereological analysis was thus performed to estimate the microvascular L_v_ (Table 1).

**Figure 2:**
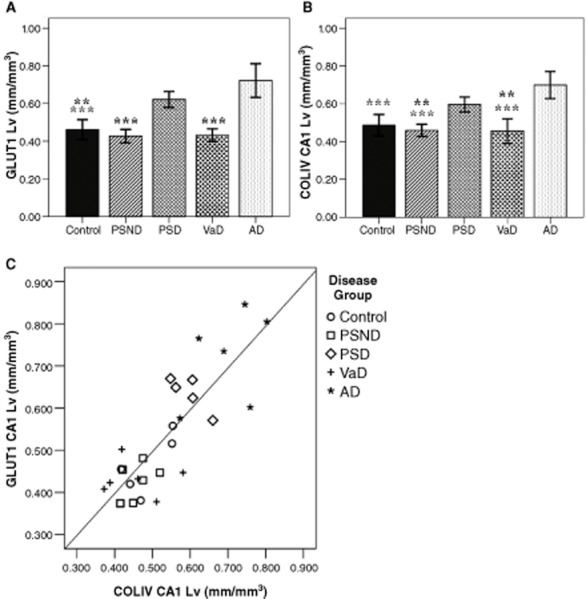
Mean L_v_ values of GLUT1 and COL4 immunostained microvessels. Both markers (**A** and **B**) showed significant increases in L_v_ in AD and PSD groups in CA1 region. Significance: **P ≤ 0.01 and ***P ≤ 0.001, different means against AD (grey), PSD (black) and both AD and PSD (black bold). Results for CA2 region with no significant changes are not shown. (**C**) The correlation between L_v_ of GLUT1 and L_v_ of COL4 immunostaining in CA1 showing internal consistency of measurement (*r*^2^ = 0.687, *P* = 0.000). Key: AD, Alzheimer's disease; controls, PSND, nondemented post-stroke subjects; PSD, post-stroke dementia; and VaD, vascular dementia.

The L_v_ results were normally distributed for all antibodies used in both the CA1 and CA2 regions (*P* > 0.05). GLUT1 L_v_, was significantly increased in the CA1 (*P* ≤ 0.001, *F* = 23.28). Significant increases in mean L_v_ in AD compared with all groups, except PSD (Figure 2**A**). There was no significant difference in L_v_ in the CA2 region (*P* = 0.459, *F* = 0.94).

COL4 L_v_ followed a similar pattern to GLUT1 changes, predominantly labelling capillaries (Figure 1). In the CA1, differences were apparent between groups (*P* ≤ 0.001, *F* = 14.92). A significant increase in L_v_ was observed between AD, compared with controls, PSND and VaD. L_v_ was significantly increased in PSD cases, compared with PSND and VaD. Similarly, in the CA2, as with GLUT1 Lv, there were no significant differences in COL4 (*P* = 0.443, *F* = 0.98).

As expected, SMA L_v_ was lower in all groups, compared with other vascular markers, due to its specificity for larger vessels (e.g. arterioles). Significant differences were apparent between the groups in the CA1 (*P* = 0.004, *F* = 5.22), where increases were observed in AD, compared with controls (AD mean L_v_ = 0.118 mm/mm^3^ *vs.* controls mean L_v_ = 0.058 mm/mm^3^, *P* = 0.027) and VaD (mean L_v_ = 0.0475 mm/mm^3^, *P* = 0.004). No significant difference was observed in CA2 (*P* = 0.959, *F* = 0.15). SMA staining had a greater coefficient of error (CE) values in all CA fields due to the relative low vessel density of arterioles. We found no correlation between L_v_ and age of subject, *post mortem* delay or length of tissue fixation.

The robustness of our L_v_ findings were verified by the strong correlation between GLUT1 and COL4 in the CA1 (*r*^2^ = 0.687, *P* = 0.000). This indicated internal consistency of the results obtained for individual microvascular L_v_ (Figure 2**C**). Thus, changes in L_v_ in the CA1 and lack of those in CA2 were reflected in both microvascular elements and profiles across all groups. The changes in either marker were not correlated with any of the clinical or psychometric measures including MMSE and CAMCOG scores (data not shown).

We also performed analysis in the EC region within the same tissue sections to ascertain microvascular density changes in the neocortical region connected to the hippocampal formation. Given the similarity in expression between GLUT1 and COL4 found in the CA1, only GLUT1 L_v_ was analysed in the EC. We observed increases in AD cases, compared with PSND (*P* = 0.004). Additionally, PSD cases had significantly lower L_v_ than AD (*P* = 0.016). PSND cases had significantly lower L_v_, compared with controls (*P* = 0.015).

### Measurement of microvessel diameters

**Figure 3:**
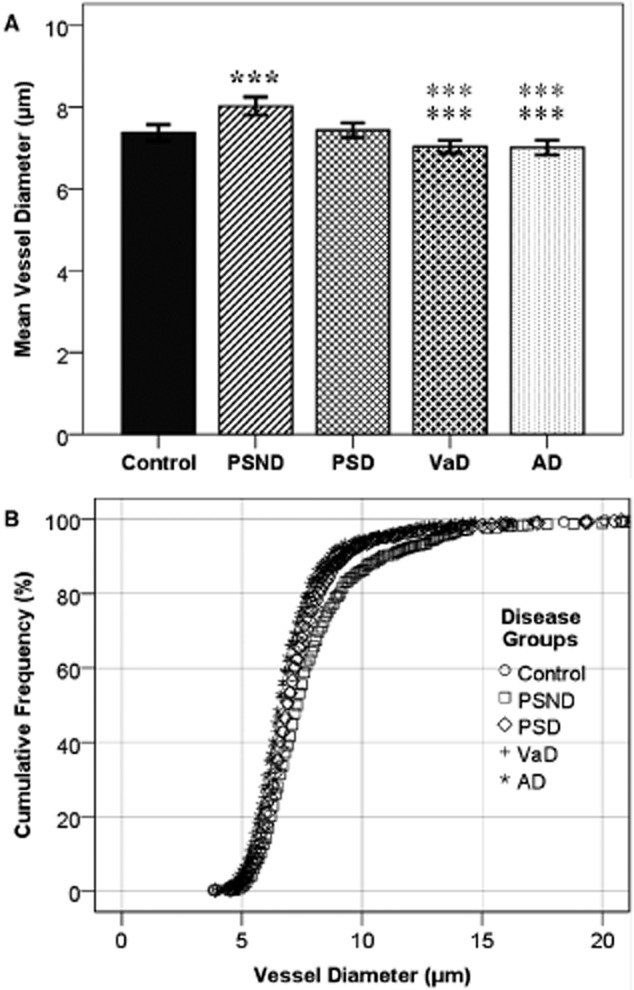
Microvessel diameters in PSD and AD *vs.* controls. (**A**) To standardize comparisons between disorders, due to increased microvessel densities in AD and PSD, the first 100 vessels measured in each case were combined to calculate the mean vessel diameter in each group. ***P ≤ 0.001. Bold black asterisks indicate significance between PSND and all other groups. Black asterisks indicate significant difference to PSD and grey asterisks show difference to controls. Bars represent 2SEM. (**B**) Cumulative frequency of increasing vessel diameter between groups. The analysis allows to identify general trends in microvascular changes within the population samples. Significant differences were found between PSND against controls, VaD, AD and PSD subjects (Table 2). Abbreviations and key to symbols: AD, Alzheimer's disease; PSND, nondemented post-stroke subjects; PSD, post-stroke dementia; and VaD, vascular dementia.

A total of 4082 microvessels were measured from all groups collectively for vessel diameter analysis. However, as there were differences in L_v_ between groups, the first 100 microvessels randomly selected in each case were analysed and combined to calculate the mean vessel diameter for each group (Figure 1). AD and VaD groups exhibited the narrowest mean diameter (7.01 and 7.04 μm respectively). The widest mean diameter was found in the PSND (8.03 μm). However, controls and PSD had similar mean diameters (7.47 and 7.44 μm respectively). After demonstrating that the data were nonparametric, we observed a significant difference between PSND and all other groups (*P* = 0.000 for all groups). A significant difference was also found between controls and PSD, compared with AD and VaD (*P* = 0.000 in all cases, Figure 3**A**). The group results were expressed in term of increasing diameter as cumulative frequency (Figure 3**B**). Significant differences were found between PSND compared with all other groups except VaD using χ^2^ distribution analysis (Table 2).

**Table 2:** Group differences between cumulative frequency distribution of vessel diameter

	Controls	PSND	PSD	VaD	AD
Controls		49.11 (*P* ≤ 0.001)	3.51 (N/S)	63.56 (*P* ≤ 0.001)	0.535 (N/S)
PSND	49.11 (*P* ≤ 0.001)		23.74 (*P* ≤ 0.001)	1.49 (N/S)	55.24 (*P* ≤ 0.001)
PSD	3.51 (N/S)	23.74 (*P* ≤ 0.001)		34.90 (*P* ≤ 0.001)	6.12 (*P* = 0.013)
VaD	63.56 (*P* ≤ 0.001)	1.49 (N/S)	34.90 (*P* ≤ 0.001)		69.89 (*P* ≤ 0.001)
AD	0.535 (N/S)	55.24 (*P* ≤ 0.001)	6.12 (*P* = 0.013)	69.89 (*P* ≤ 0.001)	

Chi^2^ (χ^2^) distribution values and significant differences between the cumulative frequencies of vessel diameter. Analysis was performed using distribution analysis, with presumed distribution three parameter lognormal.

N/S denotes no significant difference.

## Discussion

Recent advances have shown that cerebral microvascular pathology is associated with age-related cognitive decline 36–38. Changes in microvessel morphology, including thinning 17 and increased tortuosity 35,39, have been described in dementia. Our results augment previous findings linking vascular pathology with cognitive decline by showing increased microvascular density in the CA1 in PSD, compared with PSND, in both an endothelial cell (GLUT1) and basement membrane (COL4) markers. Such increases in L_v_ may be a reactive response, whereby increases in the perfusion surface between blood and brain result in remodelling of microvessels within a hypoxic environment. This interpretation does not contradict the notion that microvessel structural changes may reflect increased tortuosity in PSD subjects. Our results thus suggest that one factor that contributes to why some post-stroke survivors succumb to dementia and others do not relates to the observed sum total of the hippocampal microvascular changes in PSD and PSND subjects. These are likely acquired over a period of time from baseline 3 months after their stroke and found at death in those who developed incident dementia compared with those who did not.

We also demonstrated significant increases in L_v_ in AD cases when compared against controls, PSND and VaD in the CA1 region, and with PSND and PSD in the EC region. This may relate to the presence of different components of AD pathology (see Supplementary data and Table S1), including amyloid plaques and neurofibrillary tangles 40. However, our findings are consistent with previous studies showing an increase in L_v_ COL4 staining in the CA1 16, as well as the temporal cortex 15 of AD cases. Our observations, which indicate no significant differences in the CA2 region, suggest that the increases in L_v_ are regionally specific and can be attributed to increased vulnerability caused by AD pathology, or as a result of hypoperfusion, in the CA1 and EC. This appears to corroborate previous findings 41, which have indicated a selective reduction in capillary density within the CA1, but not the CA3 region, albeit after acute ischaemic insults.

Consistent with our previous work 42,43, GLUT1 staining was not continuous along the vessel, unlike COL4 (Figure 1). This implied that the numbers of inter-sections recorded between the probe and the vessel may not have fully corresponded with GLUT-1 expression. We thus counteracted this issue by visualizing vessels with a haematoxylin counterstain. Nevertheless, intersections were only recorded when other portions of the vessel were positively stained for GLUT1. The irregular immunostaining of endothelial cells with GLUT1 along the vessel is likely to be due to cellular damage 43. It is possible that the GLUT1-negative profiles, seen only with haematoxylin counterstaining, reflect reduced GLUT1 protein in cases with dementia 44–46.

We assessed SMA immunoreactivity, which predominately labelled smooth muscle cells within perforating branches of the hippocampal arterioles in CA1 and CA2. A significant increase in SMA L_v_ was found in CA1 in AD compared with controls and VaD; however, no difference was found between PSND and PSD.

Previous neuroimaging studies have suggested that medial temporal lobe atrophy occurs in AD 47, as well as in PSD 48. Thus, it could be argued that the observed increase in L_v_ in AD and PSD is an indicator of hippocampal atrophy or even tissue shrinkage, whereby loss of tissue reduces the distance between existing microvascular profiles 35. Valid comparisons can be made of *post mortem* tissue as all the tissues were treated in a standardized manner so any difference would be disease specific. In a related study 10, we found that hippocampal neuronal volume was decreased across all demented groups, thereby suggesting that hippocampal atrophy is not a unique finding to AD but is also apparent in PSD and VaD. Irrespective of such findings, the outcomes of the current study did not concur with the notion that atrophy was the single factor leading to an increase in L_v_, as we found a significant decrease in L_v_ in PSD cases in the EC compared with AD and no change in L_v_ in the CA1. If one hypothesizes that atrophy is the only factor leading to increased L_v_ then one would expect similar findings in both regions. Additionally, there was no change in L_v_ observed in VaD cases, compared with control or PSND subjects. As atrophy could not be measured in the cases included in this study (MRI scans were not performed in all subjects who came to autopsy), it cannot be ruled out as a confounding factor. However, if the morphological changes were related to atrophy within the whole hippocampal formation or medial temporal lobe one would predict an increase in microvessel L_v_, compared with PSND subjects in all regions and including VaD cases 49.

A technical limitation in this study was lack of availability of the whole reference volume (the hippocampus and EC) for cutting and sampling. The tissue was obtained from predefined blocks and as a result only one block per case was available for sampling. As a result of these inherent issues, we were unable to estimate volume of the hippocampus, meaning it was not possible to convert L_v_ into total length per structure (that is, the hippocampus). As L_v_ is a measure that relies on the relationship between the numerator (in this case, the number of vessel intersections) and the denominator (the background neuronal tissue), this leaves open the possibility of ‘reference trap’ bias from tissue shrinkage as, if one assumes that the density of a component within a structure acts as a proxy for its total number, one must also make the assumption that the reference volume of the structure itself must remain unchanged across the groups measured. Although we cannot rule out the effects of shrinkage, all sections were processed identically and assessment of section thickness revealed no significant differences between groups making group effects unlikely. While only six cases were analysed in each group, retrospective power analysis showed that there is a significant number of cases per group to obtain a high level of significant power.

Consistent with previous studies 14,17, a significant decrease in vessel diameter was identified in AD and VaD. Possible string vessels 36,43 were often observed but not quantified. These results may explain the commonly associated reduced CBF previously described in dementia and GLUT1 protein density 35. A number of studies have suggested that amyloid β may play a direct role in increasing vasoconstriction 50 or that nitric oxide, a natural vasodilator, is altered in dementia 51. Another study has suggested that the vasoconstrictor endothelin-1 (ET-1) may play a role in both AD and VaD. However, changes in the mRNA of the endothelin converting enzyme (ECE-1) were small and increases found in ET-1 are most likely be caused by Aβ mediated up-regulation of the converting enzymes 52,53. Furthermore, inhibiting angiotensin II, a potent vasoconstrictor may improve cognitive function in AD and VaD by ameliorating hypotension. However, angiotensin II is also involved in inhibiting the release of acetylcholine and the up-regulation of inflammatory response 54,55. Patients with AD are less likely to have been prescribed angiotensin II receptor blockers compared with age matched nondemented controls 56, which may affect vessel diameter. Microvessel diameter was significantly increased in PSND, compared with controls and no significance difference was found between PSD and controls. The results outlined may thus suggest either that vessels in PSND are more vasoreactive and responsive to their environment after stroke and undergo re-modelling (unlike PSD cases), or, alternatively, that the vessel diameter may not be related to cognitive function after stroke.

Cumulative frequency analysis showed that there were significant differences in distribution analysis of increasing vessel diameter between the groups, suggesting variations in subpopulations of hippocampal microvessels. In the PSND group, vessel diameters were significantly wider than all the other groups. Cumulative frequency analysis suggested that there were greater proportions of vessels with a diameter (between approximately 7 and 12 μm), compared with the other groups, and that the mean diameter increase was not caused by analysing a larger number of large vessels, including arterioles. This could mean that the PSND group had microvasculature that is more adaptable in response to hypoperfusion. Distribution analysis showed that there was a significant difference between the PSND group and all other groups, except VaD. This suggests a significant variation in the composition of microvasculature across all groups.

In sum, our results show an increase in microvascular L_v_ in PSD and AD and, furthermore, that microvessels were significantly wider in PSND cases. Moreover, there were differential microvascular changes across the regions of the hippocampus, with significant differences only found in the CA1 region. A significant increase was also observed in AD cases compared with both PSND and PSD in the EC. Given that there is an increase in L_v_ in PSD cases compared with PSND, but that they exhibit similar but minimal neurodegenerative pathology, the microvascular changes found may be marker for hypoperfusion in post-stroke survivors. While there were no significant differences in L_v_ between PSND and VaD subjects, it is possible that the microvasculature attributes are similar in PSD and AD in the hippocampus (but not EC) but that different mechanisms occur in VaD. A significant increase in mean vascular diameter (between 7 and 12 μm) was also found in PSND cases, compared with other groups; however, there was no significant increase in the proportion of larger vessels, that is, arterioles. Furthermore, there was no significant difference in vessel diameter between controls and PSD suggesting that microvessels may not possess the ability to adapt to their environment and thus remained unaltered. It has been widely hypothesized that the decrease in diameter in AD and VaD would reduce CBF and increase brain hypoperfusion. This suggests that the increases in the proportion of small narrower vessels in AD and PSD, when compared with PSND, may be an indicator of new microvascular loops via angiogenic processes 23, or increased twisting of existing profiles 36. However, alternative factors, such as atrophy, cannot be completely dismissed as a reason for the increases in microvascular density found in AD and PSD.

## Supporting Information

Table S1 The relationship of L_v_ with the burden of neuropathology.

## References

[1] Hofman A, Ott A, Breteler M, Bots M, Slooter A, van Harskamp F, van Duijn C, Van Broeckhoven C, Grobbee D (1997). Atherosclerosis, apolipoprotein E, and prevalence of dementia and Alzheimer's disease in the Rotterdam Study. Lancet.

[2] Snowdon DA, Greiner LH, Mortimer JA, Riley KP, Greiner PA, Markesbery W (1997). Brain infarction and the clinical expression of Alzheimer disease. The Nun Study. JAMA.

[3] Skoog I, Lernfelt B, Landahl S, Palmertz B, Andreasson LA, Nilsson L, Persson G, Oden A, Svanborg A (1996). 15-year longitudinal study of blood pressure and dementia. Lancet.

[4] Elias M, Sullivan L, Elias P, Vasan R, D'Agostino SR, Seshadri S, Au R, Wolf P, Benjamin E (2006). Atrial fibrillation is associated with lower cognitive performance in the Framingham offspring men. J Stroke Cerebrovasc Dis.

[5] Pasquier F, Boulogne A, Leys D, Fontaine P (2006). Diabetes mellitus and dementia. Diabetes Metab.

[6] Kalaria RN (2010). Vascular basis for brain degeneration: faltering controls and risk factors for dementia. Nutr Rev.

[7] Schuff N, Matsumoto S, Kmiecik J, Studholme C, Du A, Ezekiel F, Miller B, Kramer J, Jagust W, Chui H, Weiner M (2009). Cerebral blood flow in ischemic vascular dementia and Alzheimer's disease, measured by arterial spin-labeling magnetic resonance imaging. Alzheimers Dement.

[8] Firbank MJ, He J, Blamire AM, Singh B, Danson P, Kalaria RN, O'Brien J (2011). Cerebral blood flow by arterial spin labeling in poststroke dementia. Neurology.

[9] de la Torre JC (2000). Critically attained threshold of cerebral hypoperfusion: the CATCH hypothesis of Alzheimer's pathogenesis. Neurobiol Aging.

[10] Gemmell E, Bosomworth H, Allan L, Hall R, Khundakar A, Oakley A, Deramecourt V, Polvikoski T, O'Brien J, Kalaria R (2012). Hippocampal neuronal atrophy and cognitive function in delayed poststroke and aging-related dementias. Stroke.

[11] Buee LUC, Hof PR, Delacourte A (1997). Brain microvascular changes in Alzheimer's disease and other dementias. Ann N Y Acad Sci.

[12] Kitaguchi H, Ihara M, Saiki H, Takahashi R, Tomimoto H (2007). Capillary beds are decreased in Alzheimer's disease, but not in Binswanger's disease. Neurosci Lett.

[13] Paris D, Townsend K, Quadros A, Humphrey J, Sun J, Brem S, Wotoczek-Obadia M, DelleDonne A, Patel N, Obregon DF, Crescentini R, Abdullah L, Coppola D, Rojiani AM, Crawford F, Sebti SM, Mullan M (2004). Inhibition of angiogenesis by Abeta peptides. Angiogenesis.

[14] Bell MA, Ball MJ (1981). Morphometric comparison of hippocampal microvasculature in ageing and demented people: diameters and densities. Acta Neuropathol (Berl).

[15] Richard E, van Gool W, Hoozemans J, van Haastert E, Eikelenboom P, Rozemuller A, van de Berg W (2010). Morphometric changes in the cortical microvascular network in Alzheimer's disease. J Alzheimers Dis.

[16] Schwartz E, Wicinski B, Schmeidler J, Haroutunian V, Hof PR (2011). Cardiovascular Risk factors affect hippocampal microvasculature in early AD. Transl Neurosci.

[17] Bouras C, Kovari E, Herrmann FR, Rivara CB, Bailey TL, von Gunten A, Hof PR, Giannakopoulos P (2006). Stereologic analysis of microvascular morphology in the elderly: Alzheimer disease pathology and cognitive status. J Neuropathol Exp Neurol.

[18] Kalaria RN, Cohen DL, Premkumar DRD, Nag S, LaManna JC, Lust WD (1998). Vascular endothelial growth factor in Alzheimer's disease and experimental cerebral ischemia. Mol. Brain Res.

[19] Grammas P, Tripathy D, Sanchez A, Yin X, Luo J (2011). Brain microvasculature and hypoxia-related proteins in Alzheimer's disease. Int J Clin Exp Pathol.

[20] Thirumangalakudi L, Samany PG, Owoso A, Wiskar B, Grammas P (2006). Angiogenic proteins are expressed by brain blood vessels in Alzheimer's disease. J Alzheimers Dis.

[21] Desai B, Schneider J, Li JL, Carvey P, Hendey B (2009). Evidence of angiogenic vessels in Alzheimer's disease. J Neural Transm.

[22] Paris D, Patel N, DelleDonne A, Quadros A, Smeed R, Mullan M (2004). Impaired angiogenesis in a transgenic mouse model of cerebral amyloidosis. Neurosci Lett.

[23] Biron K, Dickstein D, Gopaul R, Jefferies W (2011). Amyloid triggers extensive cerebral angiogenesis causing blood brain barrier permeability and hypervascularity in Alzheimer's disease. PLoS ONE.

[24] Meyer EP, Ulmann-Schuler A, Staufenbiel M, Krucker T (2008). Altered morphology and 3D architecture of brain vasculature in a mouse model for Alzheimer's disease. Proc Natl Acad Sci U S A.

[25] Hooijmans CR, Graven C, Dederen PJ, Tanila H, van Groen T, Kiliaan AJ (2007). Amyloid beta deposition is related to decreased glucose transporter-1 levels and hippocampal atrophy in brains of aged APP/PS1 mice. Brain Res.

[26] Wu W, Brickman A, Luchsinger J, Ferrazzano P, Pichiule P, Yoshita M, Brown T, DeCarli C, Barnes C, Mayeux R, Vannucci S, Small S (2008). The brain in the age of old: the hippocampal formation is targeted differentially by diseases of late life. Ann Neurol.

[27] Mouton PR, Gokhale AM, Ward NL, West MJ (2002). Stereological length estimation using spherical probes. J Microsc.

[28] Yamamoto Y, Ihara M, Tham C, Low R, Slade J, Moss T, Oakley A, Polvikoski T, Kalaria R (2009). Neuropathological correlates of temporal pole white matter hyperintensities in CADASIL. Stroke.

[29] Burke M, Oakley AE, Slade JY, Yamamoto Y, Khundakar A, Kalaria RN (2011). Assessment of Hippocampal microvasculature in elderly demented patients. Neuropathol Appl Neurobiol.

[30] Allan LM, Rowan EN, Firbank MJ, Thomas AJ, Parry SW, Polvikoski TM, O'Brien JT, Kalaria RN (2012). Long term incidence of dementia, predictors of mortality and pathological diagnosis in older stroke survivors. Brain.

[31] Ballard C, Stephens S, Kenny RA, Kalaria R, Tovee M, O'Brien J (2003). Profile of neuropsychological deficits in older stroke survivors without dementia. Dement Geriatr Cogn Disord.

[32] Ballard C, Rowan E, Stephens S, Kalaria R, Kenny RA (2003). Prospective follow-up study between 3 and 15 months after stroke: improvements and decline in cognitive function among dementia-free stroke survivors >75 years of age. Stroke.

[33] Kalaria RN, Kenny RA, Ballard CG, Perry R, Ince P, Polvikoski T (2004). Towards defining the neuropathological substrates of vascular dementia. J Neurosci.

[34] Thal D, Rub U, Orantes M, Braak H (2002). Phases of A beta-deposition in the human brain and its relevance for the development of AD. Neurology.

[35] Kalaria RN (1996). Cerebral vessels in ageing and Alzheimer's disease. Pharmacol Ther.

[36] Brown W, Thore C (2011). Review: Cerebral microvascular pathology in aging and neurodegeneration. Neuropathol Appl Neurobiol.

[37] Hunter JM, Kwan J, Malek-Ahmadi M, Maarouf CL, Kokjohn TA, Belden C, Sabbagh MN, Beach TG, Roher AE (2012). Morphological and pathological evolution of the brain microcirculation in aging and Alzheimer's disease. PLoS ONE.

[38] van Dijk E, Prins N, Vrooman H, Hofman A, Koudstaal P, Breteler M (2008). Progression of cerebral small vessel disease in relation to risk factors and cognitive consequences. Stroke.

[39] Kalaria RN, Kroon SN, Grahovac I, Perry G (1992). Acetylcholinesterase and its association with heparan sulphate proteoglycans in cortical amyloid deposits of Alzheimer's disease. Neuroscience.

[40] Kawai M, Kalaria RN, Harik SI, Perry G (1990). The relationship of amyloid plaques to cerebral capillaries in Alzheimer's disease. Am J Pathol.

[41] Cavaglia M, Dombrowski SM, Drazba J, Vasanji A, Bokesch PM, Janigro D (2001). Regional variation in brain capillary density and vascular response to ischemia. Brain Res.

[42] Kalaria RN, Kroon SN (1992). Expression of leukocyte antigen CD34 by brain capillaries in Alzheimer's disease and neurologically normal subjects. Acta Neuropathol (Berl).

[43] Kalaria RN, Hedera P (1995). Differential degeneration of the cerebral microvasculature in Alzheimer's disease. Neuroreport.

[44] Kalaria R, Harik S (1989). Reduced glucose transporter at the blood-brain barrier and in cerebral cortex in Alzheimer disease. J Neurochem.

[45] Simpson L, Chundu K, Davies-Hill T, Honer W, Davies P (1994). Decreased concentrations of GLUT1 and GLUT3 glucose transporters in the brains of patients with Alzheimer's disease. Ann Neurol.

[46] Liu Y, Liu F, Grundke-Iqbal I, Iqbal K, Gong C (2009). Brain glucose transporters, O-GlcNAcylation and phosphorylation of tau in diabetes and Alzheimer's disease. J Neurochem.

[47] Frisoni G, Fox N, Jack C, Scheltens P, Thompson P (2010). The clinical use of structural MRI in Alzheimer disease. Nat Rev Neurol.

[48] Firbank M, Burton E, Barber R, Stephens S, Kenny R, Ballard C, Kalaria R, O'Brien JT (2007). Medial temporal atrophy rather than white matter hyperintensities predict cognitive decline in stroke survivors. Neurobiol Aging.

[49] Firbank MJ, Allan LM, Burton EJ, Barber R, O'Brien JT, Kalaria RN (2011). Neuroimaging predictors of death and dementia in a cohort of older stroke survivors. J Neurol Neurosurg Psychiatry.

[50] Niwa K, Porter V, Kazama K, Cornfield D, Carlson G, Iadecola C (2001). A Beta peptides enhance vasoconstriction in cerebral circulation. Am J Physiol Heart Circ Physiol.

[51] Price J, Chi X, Hellermann G, Sutton E (2001). Physiological levels of amyloid induce cerebral vessel dysfunction and reduce endothelial nitric oxide production. Neurol Res.

[52] Palmer JC, Barker R, Kehoe PG, Love S (2012). Endothelin-1 is elevated in Alzheimer's disease and upregulated by amyloid-beta. J Alzheimers Dis.

[53] Palmer JC, Kehoe PG, Love S (2010). Endothelin-converting enzyme-1 in Alzheimer's disease and vascular dementia. Neuropathol Appl Neurobiol.

[54] Kehoe PG, Passmore PA (2012). The renin-angiotensin system and antihypertensive drugs in Alzheimer's disease: current standing of the angiotensin hypothesis?. J Alzheimers Dis.

[55] Wharton W, Stein JH, Korcarz C, Sachs J, Olson SR, Zetterberg H, Dowling M, Ye S, Gleason CE, Underbakke G, Jacobson LE, Johnson SC, Sager MA, Asthana S, Carlsson CM (2012). The effects of ramipril in individuals at risk for Alzheimer's disease: results of a pilot clinical trial. J Alzheimers Dis.

[56] Davies NM, Kehoe PG, Ben-Shlomo Y, Martin RM (2011). Associations of anti-hypertensive treatments with Alzheimer's disease, vascular dementia, and other dementias. J Alzheimers Dis.

