# Supplementary material for: Morphometry of the hippocampal microvasculature in post-stroke and age-related dementias
Source: Neuropathol Appl Neurobiol. 2014 Mar 13;40(3):284–95. doi: 10.1111/nan.12085 (PMC4282329; doi:10.1111/nan.12085)
Supplement: Table S1 — The relationship of Lv with the burden of neuropathology. [file nan0040-0284-SD1.doc]

**Supplementary data**

The L_v_ values were also related to pathology, specifically Alzheimer-type, including Braak staging and Thal and CERAD scores. Braak scores were correlated with L_v_ in CA1 for GLUT1 (0.540, p=0.004), COL4 (0.635, p=0.000) and SMA (0.455, p=0.020). Thal staging also showed a significant correlation in CA1 with GLUT1 (0.517, p=0.010) and COL4 (0.532 p=0.070). However, there was no correlation between L_v_ and SMA. CERAD scores were correlated with GLUT1 (0.501, p=0.015) and COL4 (0.540, p=0.008) in the CA1; a trend was also observed with SMA staining (0.406, p=0.054) (Table). There was no correlation observed between L_v_ values and any pathological substrate in the CA2.

**Supplementary Table**: **The relationship of L_v_ with the burden of neuropathology**

|  | Braak staging | | | Thal et al staging | | | CERAD scores | | |
| --- | --- | --- | --- | --- | --- | --- | --- | --- | --- |
|  | Correlation Coefficient | P value | R^2^ | Correlation Coefficient | P value | R^2^ | Correlation Coefficient | P value | R^2^ |
| GLUT1 | 0.54 | 0.004 | 0.295 | 0.517 | 0.01 | 0.235 | 0.501 | 0.015 | 0.234 |
| COL4 | 0.635 | 0.000 | 0.403 | 0.532 | 0.007 | 0.283 | 0.540 | 0.008 | 0.292 |
| SMA | 0.455 | 0.02 | 0.207 | 0.257 | 0.225 | 0.066 | 0.406 | 0.054 | 0.165 |

There were significant correlations between increasing L_v_ and neuropathological scoring criteria for Braak staging, Thal staging and CERAD scores. The L_v_ was not significantly related to the vascular pathology scores for any of the groups (not shown). Abbreviations: COL4, collagen IV; GLUT1, glucose transporter 1; SMA, α-smooth muscle actin.
